# Case report: MEK inhibitor as treatment for multi-lineage mosaic KRAS G12D-associated epidermal nevus syndrome in a pediatric patient

**DOI:** 10.3389/fneur.2024.1466946

**Published:** 2024-09-25

**Authors:** Margarita Dionysiou, Stavriani C. Makri, Shivani Ahlawat, Melike Guryildirim, Kristin W. Barañano, Mari L. Groves, Pedram Argani, Christine A. Pratilas

**Affiliations:** ^1^Division of Pediatric Oncology, Johns Hopkins University School of Medicine, Baltimore, MD, United States; ^2^Russell H. Morgan Department of Radiology and Radiological Science, Johns Hopkins University School of Medicine, Baltimore, MD, United States; ^3^Department of Neurology, Johns Hopkins University School of Medicine, Baltimore, MD, United States; ^4^Department of Pediatric Neurosurgery, Johns Hopkins University School of Medicine, Baltimore, MD, United States; ^5^Department of Pathology, Johns Hopkins University School of Medicine, Baltimore, MD, United States

**Keywords:** RASopathy, KRAS G12D, hypertrophic neuropathy, selumetinib, epidermal nevus syndrome

## Abstract

The RASopathies, collectively, are a spectrum of genetic syndromes caused by mutations in genes involved in the RAS/ mitogen-activated protein kinase (MAPK) pathway, including but not limited to *PTPN11*, *NRAS*, *KRAS*, *HRAS*, *BRAF*, and *MAP2K1*. Recognized RASopathy conditions include neurofibromatosis type 1 (NF1), Noonan syndrome, capillary malformation-arteriovenous malformation syndrome, Costello syndrome, cardiofacio-cutaneous (CFC) syndrome, LEOPARD syndrome and Legius syndrome. The RASopathies often display overlapping clinical features, presumably owing to common RAS-MAPK signaling pathway activation driving dysregulated cell proliferation. Epidermal nevus syndromes (ENS) are described as the presence of epidermal nevi, in individuals also affected by extra-cutaneous organ system involvement, and there is recent recognition of mosaic RAS mutations as molecular drivers of ENS. Currently, no curative treatments exist for RASopathy driven conditions, but rather symptom-directed management is the currently accepted standard. Here, we detail a unique case of a child exhibiting diffuse spinal nerve root hypertrophy in the context of epidermal nevus syndrome driven by molecularly confirmed *KRAS G12D* mosaicism, treated with the MEK 1/2 inhibitor selumetinib. Herein, we report the response of this patient to targeted therapy of more than two years’ duration, including stabilization of multilevel nerve root hypertrophy as well as significant improvement in epidermal nevi. While the effectiveness of MEK inhibitors such as selumetinib is established in *NF1*-associated inoperable plexiform neurofibromas, their use in managing hyperactive *KRAS*-driven epidermal nevi and hypertrophic neuropathy remains unproven, and this case, to our knowledge, is the first such case to be reported. Shared molecular dysregulation and overlapping clinical features between these conditions suggest potential for effective therapeutic application of MEK directed therapy to address a range of conditions resulting from germline and/ or mosaic expression of aberrantly regulated RAS signaling.

## Introduction

The RAS family of guanosine triphosphatases (GTPases) plays crucial roles in regulating key signaling pathways responsible for normal cellular proliferation. Three members of the RAS family—*HRAS*, *KRAS*, and *NRAS*—are recurrently altered in human tumors, typically through somatic mutations at one of four hotspot codons (G12, G13, Q61, or A146). However, in conditions collectively referred to as RASopathies, RAS mutations occur as germline or post-zygotic events, leading to either constitutional or mosaic expression of mutant hyperactive RAS. These developmental syndromes display a range of overlapping phenotypic features due to common mechanisms of RAS/MAPK pathway dysregulation (1). The spectrum of mutations in human cancer differs from that in germline RASopathies. Classical oncogenic variants, such as those affecting *KRAS* codon Gly12, are typically not tolerated in the germline. However, these variants may survive in mosaicism resulting from postzygotic mutational events, leading to congenital anomalies or syndromes different from constitutional RASopathies (2). Examples of *KRAS* mosaic syndromes include oculoectodermal syndrome (OES) (OMIM 600268), encephalo-cranio-cutaneous lipomatosis (ECCL) (OMIM 613001), and epidermal nevus syndrome (ENS) (3) and their associated syndromes, such as linear nevus sebaceous syndrome (1), phacomatosis pigmentokeratotica and cutaneous-skeletal-hypophosphatasia (CSHS) syndrome (4). The clinical phenotype of mosaic RASopathies varies depending on the affected cell type, timing of mutation, and level and nature of pathway activation (5). Recent case series have expanded the association with embryonal tumors, noting previously unreported findings like Wilms tumor and nephroblastomatosis in two individuals. Other rare findings included epilepsy, polycystic kidneys, T-cell deficiency, and multifocal lytic bone lesions (6). It is important to note that the clinical variability in RASopathies is closely related to the extent of molecular variability and heterogeneity of these disorders.

Localized hypertrophic neuropathy, a rare condition of unknown etiology, is characterized by Schwann cell proliferation with prominent-bulb formation. Evidence suggests that a subset of localized hypertrophic neuropathies might develop due to RAS/MAPK pathway dysregulation, although the exact mechanisms remain unidentified. There are several published reports of hypertrophic neuropathy in patients with RASopathies, including for instance, a 23 year-old patient with Costello syndrome and peripheral hypertrophic neuropathy with germline *KRAS* point mutation (p.K5E), and three patients with CFC syndrome in two families, one of whom, a child with gait developmental abnormalities, carried a *KRAS* point mutation (c.211 T > C, p. Tyr71His) (7, 8). Additionally, an 11 year-old boy with café-au-lait spots and nerve root thickening was found to have a *KRAS* alteration in peripheral nerve and cutaneous melanocyte specimens, but not in the blood, indicating a mosaic presentation of RASopathy (9). These cases suggest a possible common mechanism underlying nerve root thickening in patients with RASopathy and concurrent hypertrophic neuropathy.

Currently, there is no known curative therapy for RASopathy conditions; rather treatment is aimed at management of clinical manifestations. Inhibiting the RAS-MAPK pathway with MEK inhibitors has proven effective in patients with NF1-associated inoperable plexiform neurofibroma (10, 11). Over 70% of such patients show radiographic and symptomatic improvement when treated with selumetinib (12, 13). Whether the same holds true for conditions caused by hyperactive KRAS (rather than loss of NF1 tumor suppressor function) remains unproven, but the molecular relationship of these molecules and the overlapping clinical syndromes suggest that therapy principles might be safely and effectively extrapolated. This hypothesis is supported by both preclinical and anecdotal clinical evidence, including at least two similar pediatric cases of mosaic RASopathy syndromes treated with a MEK inhibitor (6, 14)—in these cases, patients were treated with off-label trametinib, an orally bioavailable allosteric MEK inhibitor, for vascular malformation and encephalocraniocutaneous lipomatosis, respectively. Herein, we describe the clinical course of a pediatric patient under our care, including a response to treatment with the MEK1/2 inhibitor selumetinib. Selumetinib resulted in the arrest of the progression of diffuse spinal nerve root hypertrophy and significantly improved the epidermal findings. Despite the limited ability to extrapolate findings from a single case, the use of selumetinib as an etiology-specific treatment for pediatric patients with hypertrophic neuropathy due to KRAS mosaic mutations warrants further investigation. This case highlights the potential of targeted therapeutic strategies in managing complex RASopathies and contributes valuable insights into the treatment of similar conditions.

### Case description

**Figure 1:**
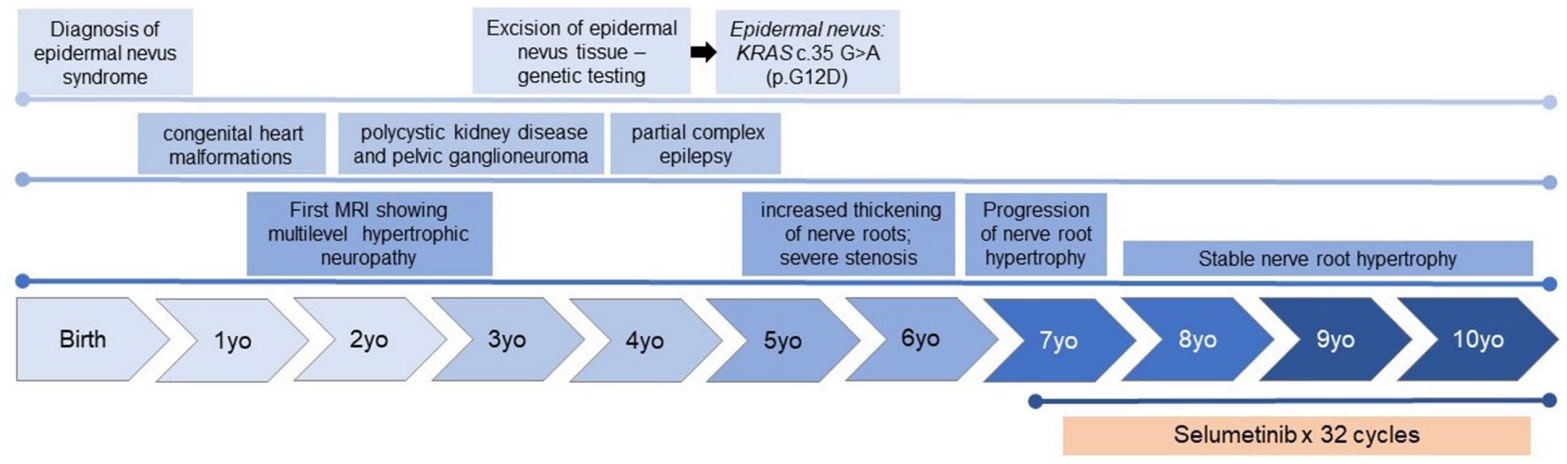
Timeline of the clinical history, including significant clinical and diagnostic findings, and key findings identified on MRI of the spine.

A 7 year-old female was referred to Pediatric Oncology for further evaluation and management of diffuse spinal nerve root enlargement with cervical cord compression, a condition that had been under surveillance for several years. She had been previously evaluated by multiple subspecialist teams for a constellation of findings, including verrucous pigmented skin lesions, congenital cardiac anomalies, polycystic kidneys, and an incidentally discovered ganglioneuroma (Figure 1).

**Figure 2:**
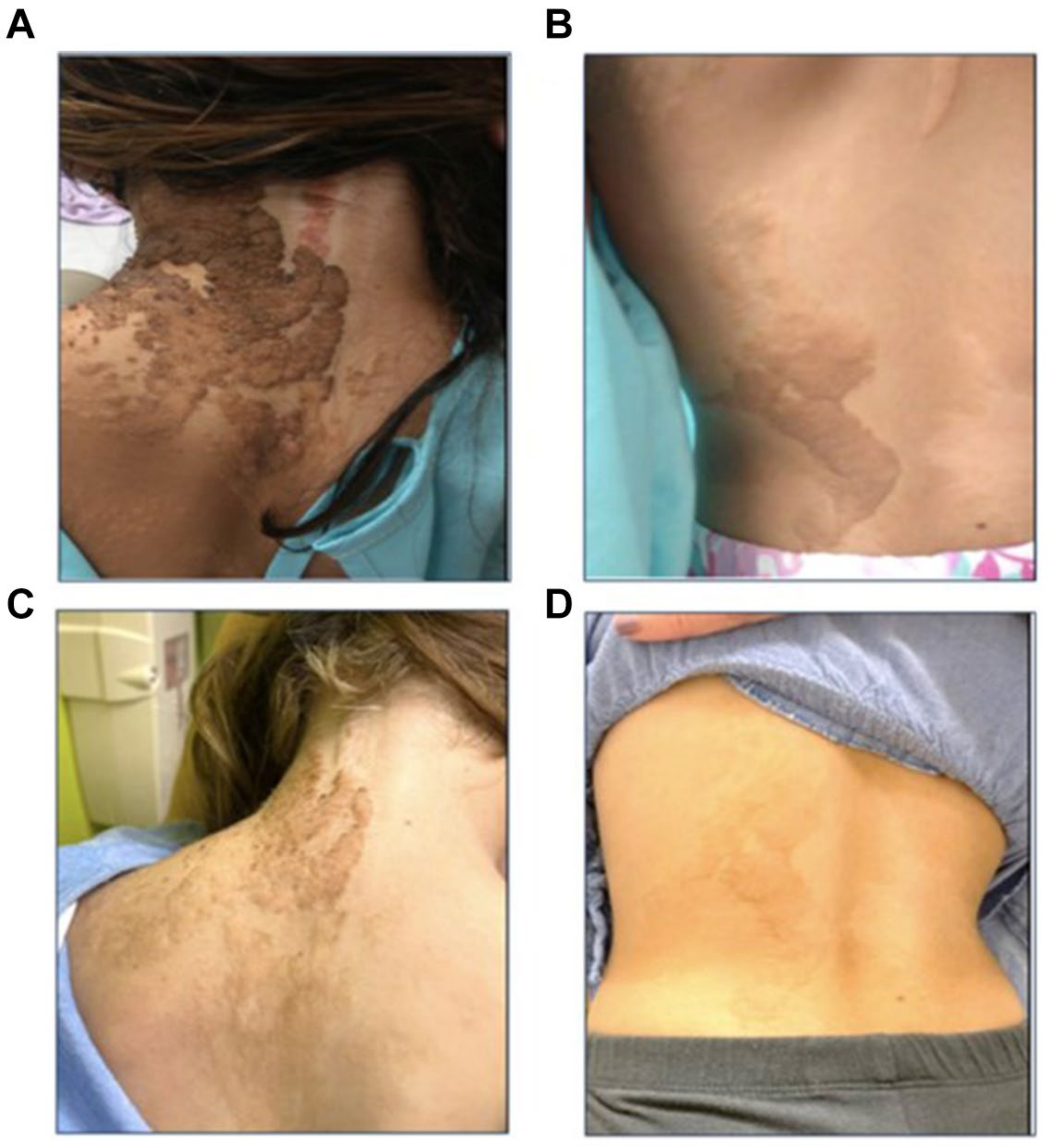
Epidermal nevi along the lines of Blaschko before **(A,B)** and during treatment with selumetinib **(C,D)**. The linear nevus primarily affects the left side of the patient’s torso **(A,B)** and the left upper extremity. There are also significant stigmata of epidermal nevus on her scalp (not shown) and deformities of the left ear helix and lobule due to the nevus. After 12 months of treatment, there is notable improvement in the affected areas, with the lesions appearing less pigmented, flatter, and smaller **(C,D)**.

**Figure 3:**
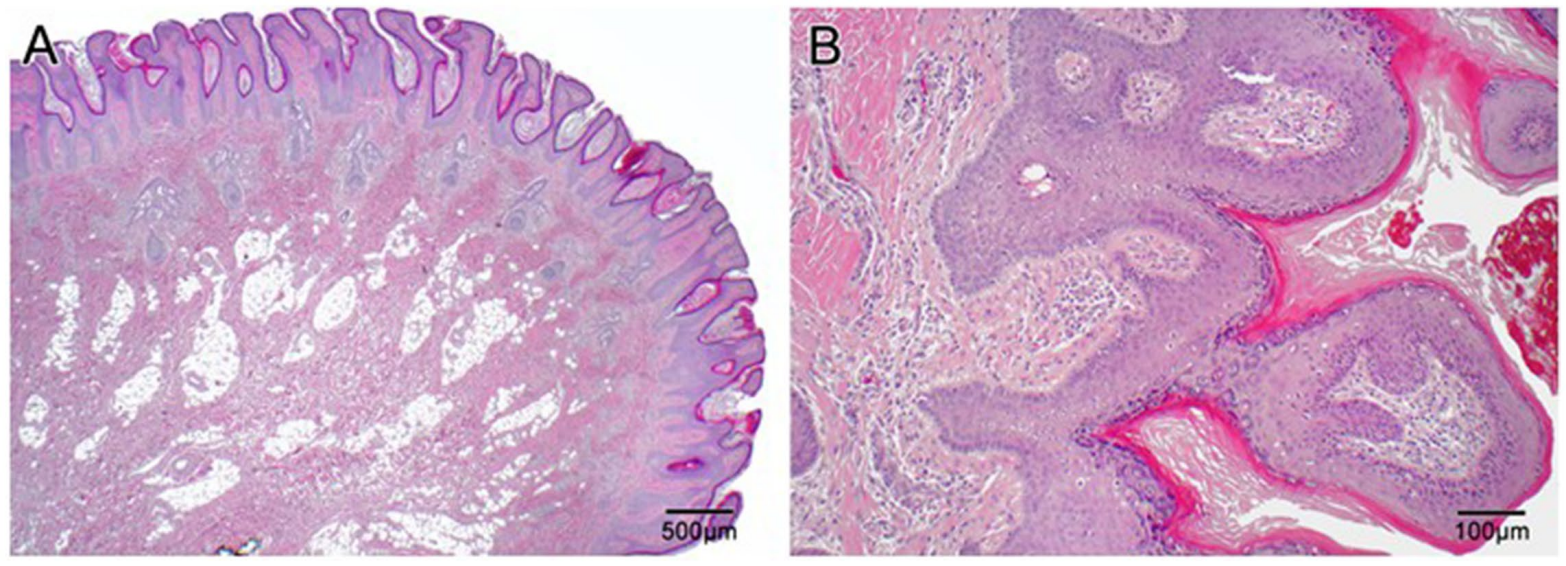
Histopathologic slides of the scalp lesion diagnosed as epidermal nevus. **(A)** Low power (2×) shows papillary epidermal hyperplasia that is both exophytic and endophytic with downward elongation of the rete into the dermis. There are aberrantly formed follicles and disorganization of the superficial connective tissue. Overall, the lesion resembles a papillomatous seborrheic keratosis. **(B)** At high power (10×) one can appreciate the papillary epidermal hyperplasia with hyperkeratosis and hypergranulosis, as well as disorganized superficial dermis.

The female, the first child of healthy, non-consanguineous parents, was born full term following an uncomplicated pregnancy. She had a normal birth weight, stature, and head circumference. A large hyperkeratotic lesion present at birth persisted into her childhood, following Blaschko’s lines across her head, neck, torso, and back, consistent with a diagnosis of epidermal nevus (Figures 2A,B). During early childhood, she was diagnosed with congenital heart malformations, benign pelvic ganglioneuroma, and polycystic kidney disease, leading to the suspicion of a RASopathy syndrome. At 3 years of age, genetic testing (including *AKT1*, *FGFR3*, *HRAS*, *KRAS*, *NF1*, *NRAS*, *PIK3CA*, *PTEN*) on the epidermal nevus tissue specimen (Figure 3) identified a *KRAS* c.35 G > A (p.G12D) mutation. This pathogenic variant, present in the epidermal nevus tissue and Schwann cells but not in blood, supported a diagnosis of *KRAS*-mediated RASopathy with mosaicism.

Beginning at the age of 18 months, an MRI of the pelvis, performed to further evaluate polycystic kidneys, incidentally revealed diffuse enlargement and T2 hyperintensity involving the lumbosacral nerve roots, dorsal ganglia, and peripheral nerves, including the sciatic and femoral nerves. A dedicated MRI of the spine showed diffuse enlargement of the T1–T2 dorsal spinal nerve roots bilaterally, with enhancement of the cauda equina nerve roots and enlargement within the foramina, particularly in the bilateral L3, L4, and sacral plexus spinal nerves. Mild flattening of the left lateral spinal cord by the enlarged left C1 spinal nerve root and mild left lateral abutment of the thecal sac by the enlarged left C2–C7 spinal nerves were also noted. At 4 years old, she was diagnosed with partial complex epilepsy, which was well-controlled with antiepileptic treatment. Apart from this, she was asymptomatic, and her psychomotor development was age appropriate.

**Figure 4:**
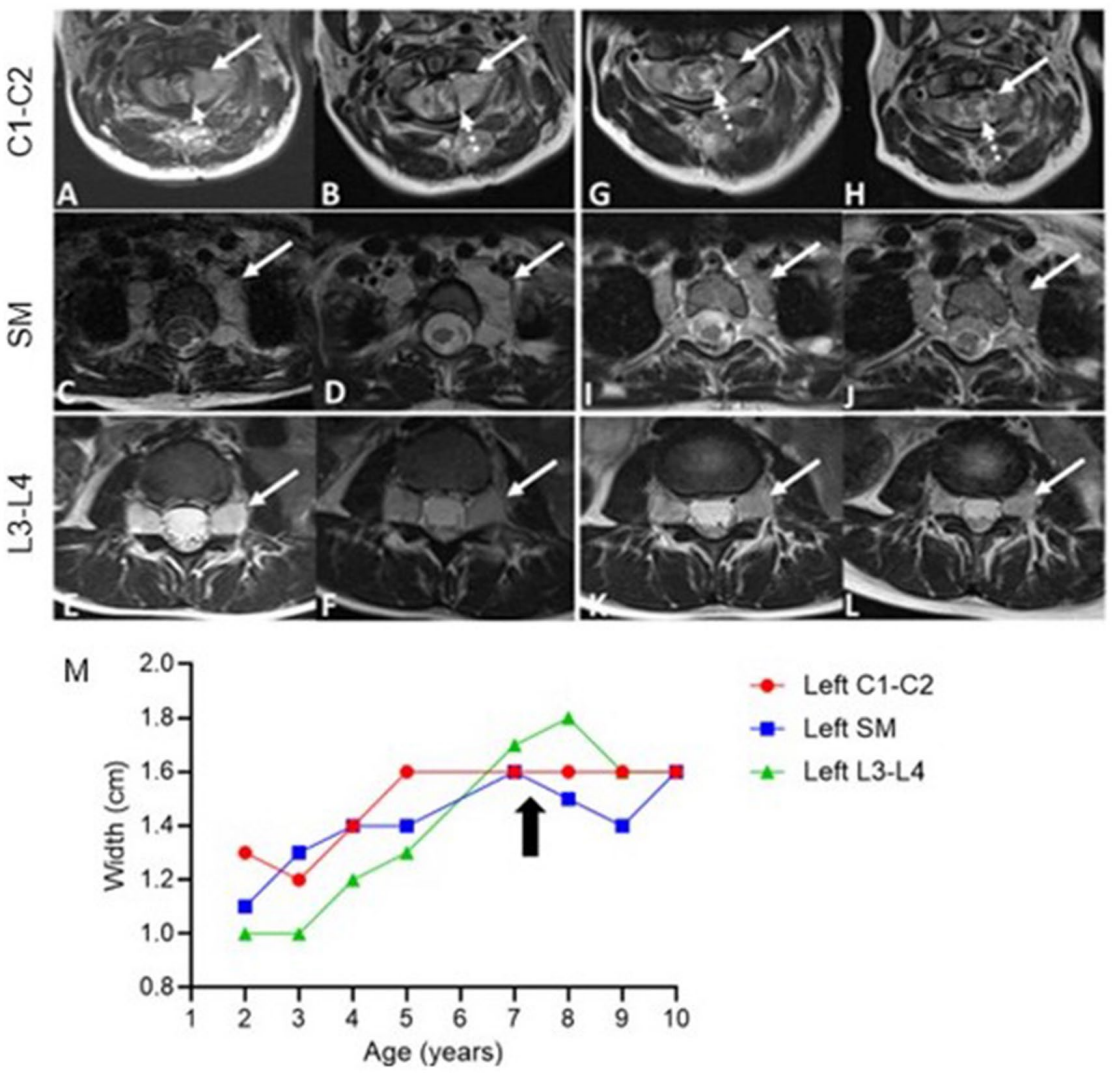
Axial T2-weighted MRI Images pre- and post-treatment. Pre-treatment **(A–F)** and post-treatment **(G–L)** images of the cervical spine (C1-C2), superior mediastinum (SM), and lumbar spine (L3-L4), Taken at 14 months **(G,I,K)** and 28 months **(H,J,L)** after initiating selumetinib. Cervical spine (C1-C2): Progressive bilateral foraminal nerve root thickening (solid arrow, **(A,B)**) with post-treatment reduction in intra-spinal thickening (dashed arrow, **(G,H)**). Superior mediastinum: Bilateral extraforaminal nerve thickening (arrows) shows initial growth before treatment **(C,D)** and stabilization post-treatment **(I,J)**. Lumbar spine (L3-L4): Persistent bilateral nerve root thickening at the neural foramina (arrows, **(E,F)**), with no significant change post-treatment **(K,L)**. Measurements of nerve root hypertrophy over time **(M)**. The graph displays the width (cm) of nerve roots at the left cervical spine (C1-C2, red), superior mediastinum (SM, blue), and left lumbar spine (L3-L4, green). The arrow marks the initiation of selumetinib treatment.

Nerve root enlargement was closely monitored over several years, with annual MRIs showing stable to mildly increased thickening of the nerve roots. At 5 years old, radiographic assessment showed a significant multilevel increase in the size of the cervical and lumbosacral nerve roots bilaterally, with severe stenosis within the upper cervical spine and partial displacement of the upper cervical cord. Subsequent radiographic assessments noted a continual increase in nerve root size. The progressive growth of hypertrophic neuropathy resulted in spinal cord compression, raising concerns for potential life-altering and devastating paralysis (Figures 4A–F). The extensive and multilevel spinal involvement precluded surgical management options.

At 7 years old, she was referred to Pediatric Oncology for further assessment and management. After carefully evaluating the potential risks and benefits, treatment with the MEK1/2 inhibitor selumetinib was initiated at the recommended pediatric dose established for patients with NF1-associated plexiform neurofibroma. Her treatment course was complicated by typical MEK inhibitor-associated dermatologic adverse events, including grade 1 acneiform dermatitis, skin dryness, eczema, and paronychia, all of which responded well to skin-directed therapies with topical emollients and occasional application of mupirocin cream. Other side effects included hair thinning and an asymptomatic mild elevation of creatine phosphokinase (CPK). Routine monitoring for drug-related toxicities, included echocardiograms and ophthalmologic exams every three months during the first year, then every 6 months thereafter, along with bone growth imaging every 6 months and magnetic resonance imaging (MRI) of the spine every 4 months during the first year, then every 6 months. Echocardiogram and ophthalmologic assessments revealed no concerning adverse events. Three months after initiating selumetinib, an MRI was performed to monitor for potential disease progression, given the experimental nature of the therapy. The imaging revealed subtle improvements in the diffuse enlargement of the cervical, upper thoracic, and lumbosacral nerve roots (Figure 4M). Follow-up MRIs showed overall stability in nerve root enlargement, with no new or worsening findings (Figures 4G–L). The most significant improvement was observed in the epidermal nevi, with lesions becoming flatter, smaller, and less pigmented in all affected areas (Figures 2C,D). Recent magnetic resonance imaging of the spine, conducted 28 months after starting treatment, continues to show stable disease. At the time of this report, the patient has been tolerating the medication well without experiencing any major side effects. She continues to be followed by a multidisciplinary team, including dermatology, nephrology, cardiology, neurology, and neurosurgery. She remains on antihypertensives due to underlying congenital heart disease, with no changes in her disease status since starting selumetinib. Her epilepsy is well controlled on levetiracetam, and she has remained seizure-free for several years. Currently, she attends middle school where she is a good student, actively participating in various physical activities, including dancing, gymnastics, and scouting.

## Discussion

Herein we describe the clinical course of a child with mosaic *KRAS*-mediated epidermal nevus syndrome (ENS), presenting with severe spinal nerve root hypertrophy, verrucous epidermal nevi, benign pelvic ganglioneuroma, and polycystic kidney disease. The most clinically threatening finding was hypertrophic neuropathy, characterized by paraspinal nerve sheath tumors, posing a risk of symptomatic cord compression. An activating *KRAS* gene mutation (*KRAS* c.35 G > A, p.G12D) identified in multiple tissue lineages is presumed to be the driver of the peripheral nervous tissue hyperproliferation. Mutations in *KRAS* such as those at Gly12 disrupt the balance of the RAS molecular switch between the GDP-bound inactive state and a GTP-bound active state, resulting in continuous activation of RAS-effector pathways and promoting cell proliferation (15).

The heterogeneity and overlapping features of the spectrum of RASopathies, as exemplified by this case, underscore the complexity of these disorders. The clinical variability closely correlates with the molecular diversity inherent to these conditions. Despite their rarity, RASopathies lead to significant morbidity and mortality, sometimes profoundly impacting early development and quality of life. The challenge in designing therapeutic trials for patients with RASopathies stems from the rarity of these conditions, the wide variability in phenotypes, the limited understanding of their natural history, and the absence of validated therapeutic endpoints (16).

Early insights into the role of the RAS/RAF/MEK/ERK signaling pathway in regulating cell proliferation established insights for the development of potent and selective MEK inhibitors. Neurofibromatosis type 1 (NF1) is a prototypic RASopathy. Through the conduct of a longitudinal cohort study and early phase clinical trials, selumetinib was identified as the first active therapy for the NF1-related peripheral nerve sheath tumors called plexiform neurofibromas (PN). As a result, selumetinib was granted breakthrough therapy designation by the FDA for the treatment of PN. Selumetinib has shown notable benefits in reducing tumor size and improving quality of life, including improvements in tumor pain intensity, mobility, and overall health-related quality of life. Importantly, these benefits contrast with the natural progression of untreated tumors, which tend to grow and worsen over time, particularly in adolescent years (17). Other MEK inhibitors have demonstrated similar clinical benefit in paitents with NF1, but selumetinib was the first to gain FDA approval for this indication and therefore was chosen for this patient. The mechanism of action of MEK inhibitors in NF1, which involves inhibiting overactive RAS/MAPK signaling due to *NF1* loss of tumor suppressor function, provides a rationale for their potential efficacy in other RASopathies with similar pathway dysregulation. Although continued radiographic evaluation of this patient’s hypertrophic neuropathy has not demonstrated objective decrease in the measured nerve root diameter, the cessation of continued growth can be considered a measure of success, given that further growth could have resulted in life-altering or life-threatening neurologic compromise including paralysis. In the case of this patient, the duration of treatment cannot be firmly established, as continued treatment will require ongoing risk–benefit assessment to ensure that the clinical benefit is established and outweighs the potential adverse effects associated with the use of MEK inhibitors.

Other RASopathy manifestations may also benefit from RAS targeted therapies. The Advancing RAS/RASopathy Therapies (ART) initiative by the NCI aims to develop therapies and prevention strategies for the clinical manifestations of the non-*NF1* RASopathies and for tumors characterized by somatic RAS mutations (16). Trametinib is a MEK inhibitor used in combination with dabrafenib, a BRAF inhibitor, to treat metastatic *BRAF*-mutated melanomas (18). In 2022, the FDA approved this combination for adults and children (aged 6 and older) with unresectable or metastatic *BRAFV600E*-mutant solid tumors, excluding *BRAFV600E*-mutant colorectal cancers. This histology-agnostic approval culminates two decades of research into BRAF mutations, their role in cancer, and the development of selective RAF and MEK inhibitors (19). Since its first FDA approval, trametinib has been tested to treat a few patients with generalized lymphatic anomaly (GLA) (20–23), hereditary hemorrhagic telangiectasia (HHT) (24), or arteriovenous malformations (AVM) (25, 26) with encouraging results. A phase II clinical trial was started in 2019, including 10 adult patients with extracranial AVMs. Preliminary results show a reduction in pain in all 10 patients and in clinical volume in 9 patients (TRAMAV; EudraCT number: 2019-003573-26). Additionally, trametinib has been successfully used in at least two pediatric cases of mosaic RASopathy syndromes, underscoring its potential across a range of RAS-driven conditions (6, 14). Of particular interest is the recent report of two adult patients who responded to treatment with sotorasib for severe KRAS G12C-related arteriovenous malformations (27).

The complexity of RASopathies, as illustrated in our case, necessitates a multidisciplinary medical approach. Given the diversity of organ systems affected and the variability in clinical manifestations (Table 1), treatment often requires a combination of therapeutic strategies tailored to individual patient needs. The effective application of a cancer drug in a non-oncologic, yet genetically related condition, opens avenues for repurposing existing medications for rare genetic disorders. It also emphasizes the importance of understanding the underlying molecular mechanisms of diseases for developing targeted therapies while encouraging further research into the broader applications of RAS-pathway targeting drugs. This case represents a significant step in addressing such complexity, highlighting the potential of MEK inhibitors as a viable option for managing specific manifestations of RASopathies, particularly those akin to the hypertrophic neuropathy observed in our patient.

**Table 1:** Published cases of KRAS-associated hypertrophic neuropathy.

Patients		Patient 1 (Bertola et al. 2007 and 2012)	Patient 2 (Stark et al. 2012)	Patient 3 (Vizcaino et al. 2020)	Patient 4 (Ando et al. 2021)	Patient 5 (Draaisma et. al. 2023)	Patient 6 (this report)
Genotype	Mutation	*KRAS* p.K5E	*KRAS* c.211T>C, p.Y71H	*KRAS* duplication c38_40dupGCG	*KRAS* c.211T>G, p.Tyr71Asp	*KRAS* in-frame deletion- insertion c.194_195ins21 (p.Ser65delins8)	*KRAS* c.35 G>A, p.G12D
	Other mutations	*NF2* c.886-15>T				WES^1^: no genetic abnormality	
Tissue tested	Peripheral blood	KRAS mutation *NF2* c.886-15>T		Not detected	KRAS heterozygous de novo mutation c.211T>G, pTyr71Asp	N/A	Not detected
	Other tissue tested	Intercostal nodule, *NF2* c.886-15>T		Peripheral nerve, *KRAS* duplication c38_40dupGCG	N/A	N/A	Epidermal nevus, *KRAS* c.35 G>A, p.G12D
				Café-au-lait (melanocytes): same alteration at a lower level			
Imaging findings		MRI: diffuse enlargement of all nerve roots of the spine, brachial plexus, radial, ulnar and median nerves, and masses on the retroperitoneum CT: ventriculomegaly	MRI: prominence of fourth ventricle	Bilateral multiple stress fractures of lower limbs, diffuse enlargement of the lumbosacral plexus and right sciatic nerve extending to the tibial, common peroneal, and deep and superficial peroneal nerves	Hypertrophy of intercostal nerve, the brachial, and lumbar plexus, a retroperitoneal tumor around the abdominal aorta and celiac artery, and hypertrophy of the sciatic and tibial nerves	Hypertrophy of right brachial plexus, right sciatic and tibial nerves, enlargement of the extraforaminal spinal nerve roots and both brachial and lumbar plexuses	Hypertrophy of lumbosacral plexus, sciatic and femoral nerves, diffuse enlargement of T1-T2 dorsal nerve roots, enlarged left C1, C2-C7 spinal nerves
Epidemiology	Age at report	23	4	11	45	10	7
	Origin	N/A	Caucasian	N/A	N/A	N/A	Caucasian
	Sex	F	M	M	F	F	F
Clinical diagnosis		Costello syndrome	CFC syndrome^2^	*KRAS*-mediated RASopathy	Noonan syndrome	Noonan syndrome with multiple lentigines (NSML)	*KRAS*-mediated RASopathy/ epidermal nevus syndrome (ENS)
Development	Developmental delay	Severe developmental delay	Motor delay speech delay	N/A	Motor delay	N/A	Speech delay
	Intellectual disability		Yes	N/A	Yes	N/A	No
Dysmorphic features		Ocular hypertelorism, down-slanting palpebral fissures, thick lips, macrocephaly, macrostomia, fleshy earlobes	Absent eyebrows, broad forehead	N/A	Low-set hairline, ocular hypertelorism, macroglossia, flat root of nose, wide wings of nose, thick lower lips, high palate, wide interdentium, malocclusion, talipes equinus contracture, hallux valgus	N/A	Mild facial asymmetry with the right side larger than the left, deformed left ear helix and lobule secondary to epidermal nevus, normal hearing
Ophthalmologic		Proptosis		N/A	Proptosis, exotropia of the left eye, no Lisch nodules	N/A	No
Neurological		Difficulty in relaxation of the hand muscles	Mild foot drop, mild wasting of both distal extremities, mild neuropathy with both demyelinating and axonal features	N/A	Amyotrophy and hypotonicity in distal limbs, vibration and proprioception impaired in distal lower limbs, hypertrophic peripheral nerve bundles in supraclavicular and popliteal fossa	Stepping gait, diffuse sensorimotor polyneuropathy with demyelinating features, burning pain in upper and lower extremities	Partial-complex epilepsy
Cutaneous		Superficial nodule in her fourth right intercostal space was palpable	N/A	Café-au-lait on the face, trunk, and extremities bilaterally	No café-au-lait, or neurofibromas	N/A	Epidermal nevus across the head, neck, back, torso. Left lateral scalp with thinner skin-colored plaque with alopecia
Lymphovascular anomalies		Lymphedema in both legs	N/A	N/A	Lymphedema in left leg	N/A	No
Musculoskeletal		Hyperextensible joints, deep palmar creases, pectus excavatum, loose skin in hands and feet, webbed neck	Bilateral ankle valgus deformity, pes planus	Right leg length discrepancy	Muscle weakness	Atrophy of the hand musculature and calf muscles, muscle weakness in upper and lower extremities	No
Cardiovascular		Hypertrophic cardiomyopathy with dysplastic pulmonary and mitral valves	Normal echocardiogram at 3 years of age	N/A	Pulmonary artery stenosis, systolic murmur (IV/VI) in the right sternum area, bilateral ventricular outlet stenosis, bilateral leg swelling	N/A	Congenital cardiac malformations (ectopic atrial tachycardia, PDA^3^, coarctation of aorta), hypertension
Genitourinary		N/A	N/A	N/A	N/A	N/A	Polycystic kidneys
Gastrointestinal		Abdominal pain, nausea	N/A	N/A	Intermittent severe abdominal pain	N/A	No
Tumors		Nasal papillomata in the right nostril, masses on retroperitoneum	N/A	N/A	Retroperitoneal tumor around abdominal aorta and celiac artery	N/A	Pelvic ganglioneuroma
Pathology		Intercostal nodule: spindle-shaped Schwann cells with symplastic degenerative changes and diffuse expression of S100 consistent with schwannoma	N/A	Tibial nerve biopsy: onion bulbs, strong pERK staining	Sural nerve biopsy: onion bulb lesions, few myelinated fibers	N/A	Scalp lesion; epidermal nevus	
MEK inhibitor		No	No	No	No	No	Selumetinib	

1 WES: Whole Exome Sequencing.

2 CFC: cardiofaciocutaneous.

3 PDA: patent ductus arteriosus.

## Data Availability

The original contributions presented in the study are included in the article/supplementary material, further inquiries can be directed to the corresponding author.
